# P-384. Cutting Through the Data: A Comparative Analysis of Surgical Site Infection Surveillance in Peripheral Vascular Bypass Surgery

**DOI:** 10.1093/ofid/ofae631.585

**Published:** 2025-01-29

**Authors:** Young Kim, Becky A Smith, Brittain A Wood, Polly W Padgette, Deverick J Anderson, Jessica Seidelman

**Affiliations:** Duke University, Durham, North Carolina; Duke University, Durham, North Carolina; Duke Infection Control Outreach Network (DICON), Morrisville, North Carolina; Duke Infection Control Outreach Network (DICON), Morrisville, North Carolina; Duke Center for Antimicrobial Stewardship and Infection Prevention, Durham, NC; Duke University School of Medicine, Durham, North Carolina

## Abstract

**Background:**

Different societies and organizations use different surgical site infection (SSI) definitions for surveillance. With multiple definitions in use, validation of surveillance findings becomes paramount, particularly for maintaining surgeon buy-in during data review and developing countermeasures. The goal of our study was to assess concordance between SSI diagnoses following peripheral vascular bypass (PVBY) surgery derived from review by a vascular surgeon (vascular) and those identified through SSI surveillance performed by the infection prevention (IP) team.

**Figure:**
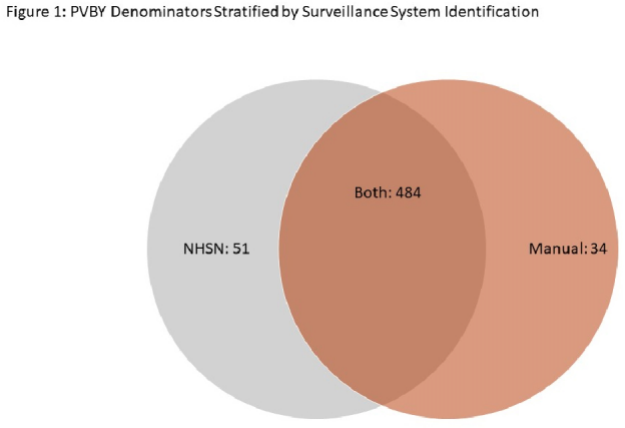

**Methods:**

The IP team performs SSI surveillance using EPIC “Bugs” tool to identify potential SSIs from NHSN selected CPT codes. EPIC then identifies cultures within the infection window period and IP reviews the case to ensure it meets SSI criteria. Conversely, the vascular surgeon reviewed every PVBY CPT coded surgery to determine if an SSI occurred. The vascular review used the Southampton Scoring System. Both surveillance systems queried PVBY surgeries from 1/1/2018 to 12/31/2022 and used a 90-day surveillance period.

**Figure:**
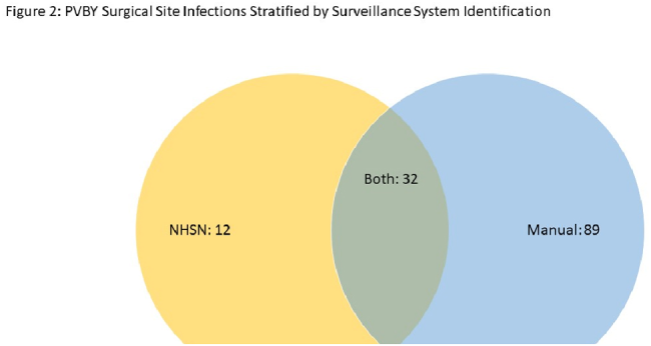

**Results:**

Among the CPT codes used for surveillances, 569 PVBY surgeries and 133 SSIs (23.4%) were identified. Both surveillance systems picked up 484 cases (85.1%), IP noted 51 (9.0%) additional cases, and vascular found the remaining 34 (6.0%) cases. (Figure 1) Among the SSIs, both datasets detected 32 SSIs (24.1%), IP recognized 12 more SSIs (9.0%) and the vascular review designated the remaining 89 (66.9%) SSIs. (Figure 2) Of the 89 SSIs picked up by the vascular review, 5 SSI surgeries were not in the IP denominator. The other 84 SSIs were included in the IP denominator, but those were not denoted as SSIs. Among the 84, the majority (64, 76%) were Southampton scores of 2 or 3. (Figure 3) Of those 84, 51 (60.7%) did not have a culture taken.

**Figure:**
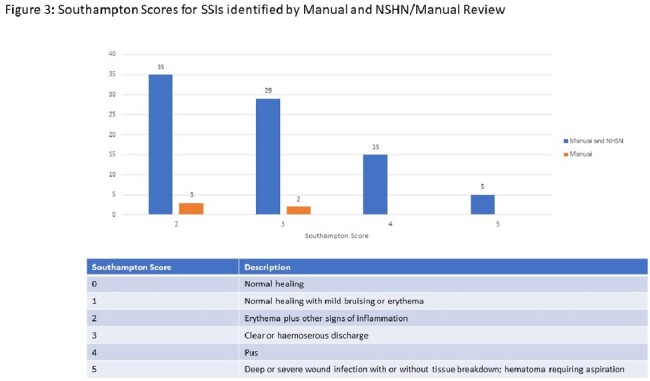

**Conclusion:**

The vascular SSI review identified 89 additional PVBY SSIs compared to the IP surveillance over the 5-year period. NHSN has limitations in PVBY SSI surveillance, particularly for superficial incisional SSIs. Although IP is not resourced to perform surveillance on every surgery, IP working with surgical teams to understand differences in surveillance can facilitate alignment needed to develop successful SSI prevention initiatives.

**Disclosures:**

**Becky A. Smith, MD**, UpToDate: royalties **Jessica Seidelman, MD, MPH**, 3M: Expert Testimony

